# Diagnostic accuracy of [^18^F]PSMA-1007 PET/CT in biochemical recurrence of prostate cancer

**DOI:** 10.1007/s00259-022-05693-0

**Published:** 2022-01-24

**Authors:** Clemens Mingels, Karl Peter Bohn, Axel Rominger, Ali Afshar-Oromieh, Ian Alberts

**Affiliations:** Department of Nuclear Medicine, Inselspital, Bern University Hospital, University of Bern, Freiburgstr. 18, 3010 Bern, Switzerland

**Keywords:** Prostate cancer, Biochemical recurrence, PET/CT, Positron emission tomography, PSMA, Prostate-specific membrane antigen

## Abstract

**Aim:**

Despite increasing use for the detection of biochemically recurrent prostate cancer (rPC), the diagnostic accuracy of positron emission tomography/computed tomography (PET/CT) with [^18^F]PSMA-1007 remains only partially investigated. The aim of this study was to determine the sensitivity (SE), specificity (SP), positive predictive value (PPV), and negative predictive value (NPV) for PC-local recurrence and metastases on a per region basis.

**Materials and methods:**

One hundred seventy-seven consecutive patients undergoing [^18^F]PSMA-1007 PET/CT for rPC were retrospectively analysed. Six body regions were defined: prostate fossa, pelvic lymph nodes (LN), retroperitoneal LN, supradiaphragmatic LN, bones, and soft tissue. A region was counted positive if at least one PSMA-positive lesion suspicious for PC was observed. Confirmation of a true-positive PSMA-avid lesion was defined as positive by histopathology, fall in serum prostate-specific antigen (PSA) (> 50%) after targeted therapy or confirmatory further CT, MRI, PET/CT, or bone scan imaging. Regions where additional imaging was able to confirm the absence of suspicious PC lesions or regions outside exclusively targeted RT with serum PSA decline (> 50%) were counted as true-negative regions. SE, SP, PPV, and NPV were calculated for all six regions.

**Results:**

The overall PET-positivity rate was 91%. Conclusive follow-up for affirmation or refutation of a PSMA-positive lesion was available for 81/152 patients on a per region basis. In this subgroup, overall sensitivity, specificity, PPV, and NPV were 95% (CI: 0.90–0.98), 89% (CI: 0.83–0.93), 86% (0.80–0.90), and 96% (CI: 0.92–0.98), respectively. On a per region basis, PPV was 97% (CI: 0.83–0.99) for local recurrence, 93% (CI: 0.78–0.98) for pelvic LN, 87% (CI: 0.62–0.96) for retroperitoneal LN, 82% (CI: 0.52–0.95) for supradiaphragmatic LN, and 79% (0.65–0.89) for bone lesions. The number of solid organ metastases (*n* = 6) was too small for an accurate statistical analysis.

**Conclusion:**

The known high PET-positivity rate of [^18^F]PSMA-1007 PET/CT in rPC was confirmed, with corresponding high (> 90%) sensitivity and NPV on a per region basis. However, overall PPV was limited (86%), particularly for bone lesions (79%), which are a potential diagnostic weaknesses when using this tracer.

**Supplementary Information:**

The online version contains supplementary material available at 10.1007/s00259-022-05693-0.

## Introduction

Radioligands to the prostate-specific membrane antigen (PSMA) have become the gold standard for the staging of primary prostate cancer (PC) [1–4] and restaging of patients with biochemical recurrence of PC (rPC), outperforming conventional imaging modalities and previous generation radiopharmaceuticals [2].

In addition to [^68^Ga]Ga-PSMA-11, a large number of alternative radioligands have become available, including but not limited to [^68^Ga]Ga-PSMA-I&T; [^68^Ga]Ga-THP-PSMA; and [^18^F]-labelled PSMA-radiotracers such as [^18^F]-rhPSMA-7, [^18^F]-DCFPyL, [18F]-JK-PSMA-7, or [^18^F]PSMA-1007 [5–8]. Currently, there is only scant evidence to support the choice of one ligand or another [9–11]. [^18^F]-labelled PSMA-radiotracers have numerous advantages over ^68^Ga-labelled ligands: [^18^F] is cyclotron produced with a longer half live and a lower positron energy compared to [^68^Ga] (0.65 MeV vs. 1.90 MeV), leading to improved spatial resolution [12]. Initial studies with [^18^F]PSMA-1007 suggest improved detection rates especially in local relapses and pelvic lymph node metastases in proximity to the urinary tract [13–17]. For these patients, [^18^F]PSMA-1007 might be of benefit [13], although the use of forced diuresis protocols with renally excreted ligands such as [^68^Ga]Ga-PSMA-11 can be an alternative [18–20], and where later acquisition time might improve lesion visibility and interpretability [21, 22]. Nevertheless, some limitations for this tracer have been reported, particularly unspecific uptake in bone lesions [23–25].

Despite widespread adoption, few studies report the diagnostic accuracy of [^18^F]PSMA-1007 in rPC. Hitherto, studies report only preliminary observations in small cohorts (*n* = 40) [16], in mixed cohorts of primary and rPC [14], thereby limiting their interpretability, or provide any verification of positive findings [5, 26]. Cognisant of the higher rate of non-specific lesions, namely in bones, an analysis of the diagnostic performance of this tracer by region is warranted. Therefore, the aim of this study was to assess the diagnostic accuracy of [^18^F]PSMA-1007 in a cohort of men undergoing PSMA-PET/CT for rPC in a clinical setting on a per patient and per region basis using a composite standard of truth (CSOT) for verification or refutation of imaging findings.

## Materials and methods

### Patient population

**Fig. 1:**
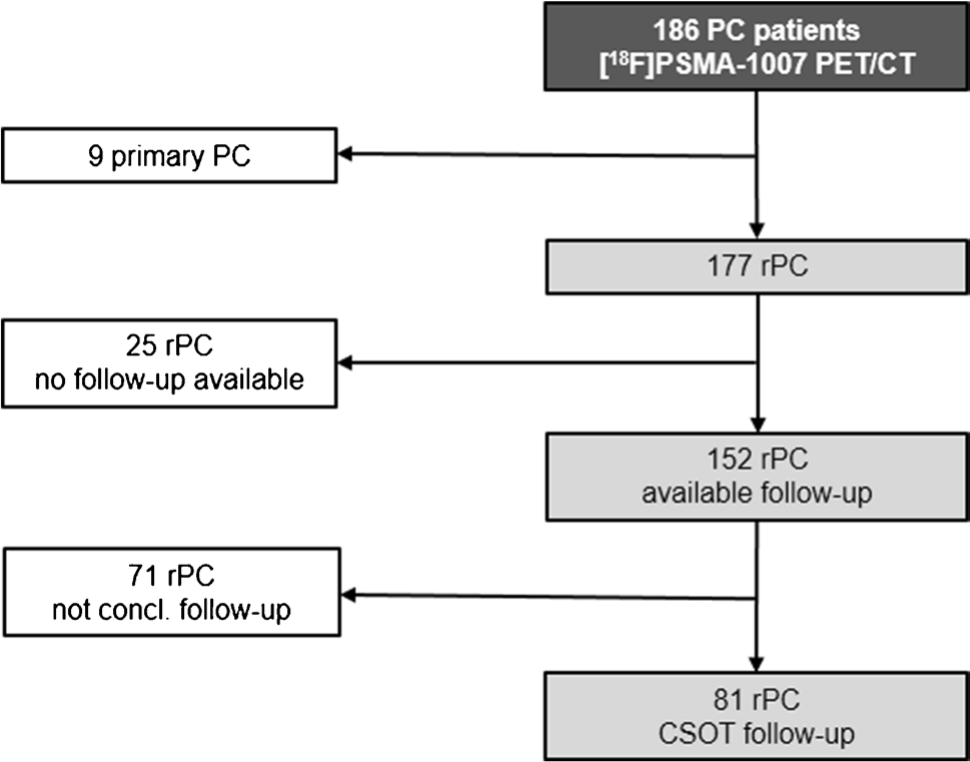
Patients screened and ex/included in the analysis. One hundred eighty-six patients underwent [^18^F]PSMA-1007 PET/CT. Nine patients with primary prostate cancer (PC) were excluded. One hundred seventy-seven patients were referred to the PET/CT with biochemical rPC. One hundred fifty-two patients with rPC had available follow-up. Lesion validation with composite standard of truth (CSOT) was possible in 81 of them

Between October 2019 and May 2020, 186 consecutive patients underwent [^18^F]PSMA-1007 PET/CT in the University Clinic for Nuclear Medicine, Inselspital, Bern. The period was chosen to enable a minimum follow-up period of 12 months. Inclusion criteria were biochemical recurrence of PC (rPC) according to the ASTRO/AUA Guideline as rising PSA value after a PSA-Nadir after definitive treatment (i.e. radical prostatectomy, targeted radiotherapy) [27, 28]. One hundred seventy-seven patients with rPC were included and retrospectively analysed (Figure 1). Patients’ characteristics are outlined in Table 1. Details of prior treatments for included patients are given in the Supplementary Materials.

**Table 1:** Patients’ characteristics of all rPC patients (*n* = 177) and of those where composite standard of truth (CSOT) follow-up was available (*n* = 81): age (median and range) (years), TNM stage (median, range; Union for International Cancer Control UICC, 8th Ed.), Gleason score (GS; median, range), and PSA (mean ± standard deviation, range) (ng/ml)

	rPC (*n* = 177)	CSOT follow-up available (*n* = 81)
Age (year) (mean, range)	71 (51–89)	70 (51–87)
T (median, range)	3 (1–4)	3 (1–4)
N (median, range)	0 (0–1)	0 (0–1)
M (median, range)	0 (0–1)	0 (0–1)
Gleason score (mean, range)	7.5 (5–10)	7.4 (5–10)
PSA value (ng/ml) (mean, SD, range)	35.07 ± 249 (0.12–3300)	62.5 ± 373 (0.14–3300)

### Radiotracer

[^18^F]PSMA-1007 was produced as previously described [29]. The [^18^F]PSMA-1007 solution was given by intravenous bolus injection (mean 245.7 ± 13.3 MBq; range 181–305 MBq).

### Imaging

All patients received regular whole-body PET/CT scans (from head to the thighs) at 120 ± 10 min after injection of [^18^F]PSMA-1007. They were investigated on either Biograph-mCT PET/CT (Siemens, Erlangen, Germany) (*n* = 90) or Biograph-VISION 600 PET/CT (Siemens, Erlangen, Germany) PET/CT (*n* = 96) scanner. The examination protocols and reconstruction algorithms used are previously published [30, 31].

### Image evaluation

Image analysis was performed using an appropriate workstation and software (SyngoVia; Siemens, Erlangen, Germany). All scans were read and dual reported by an experienced nuclear medicine physician and an experienced resident. All imaging reports were gathered and scrutinised by two experienced nuclear medicine physicians (IA and CM). If at least one PSMA-positive lesion suspicious for PC was described, the PET/CT was counted as positive. Any PSMA-positive lesions suspicious for PC were further categorised according to their location in six different body regions (local recurrence, pelvic lymph nodes (LN), retroperitoneal LN, supradiaphragmatic LN, bone lesions and soft tissue lesions). A region was counted “positive”, if at least one PSMA-positive lesion suspicious for PC was detected.

### Lesion validation per region

**Fig. 2:**
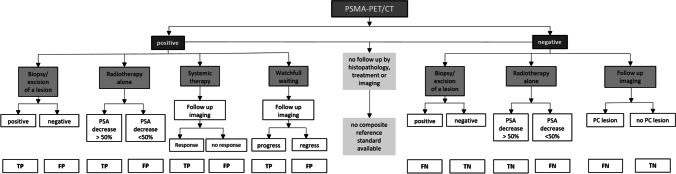
Composite standard of truth (CSOT) defined positive and negative PSMA-regions after follow-up for histopathology, radiotherapy with PSA decline, other treatments, and correlative imaging of the regions as true positive (TP), false positive (FP), true negative (TN), and false negative (FN). Patients with no follow-up imaging, treatment, or histopathology were excluded

All patients’ clinical records were scrutinised for details of follow-up that could confirm or refute the imaging findings. Correlative imaging, biopsy, or fall in serum PSA (> 50%) following exclusively targeted radiotherapy of a PSMA-positive lesion suspicious for PC were true positive (TP). The absence of PSMA-positive legions in a region where additional imaging revealed the presence of PC or where histopathology showed a positive result were counted as false negative (FN). A false-positive (FP) region was counted where a PMSA-positive lesion was observed in [^18^F]PSMA-1007 PET/CT, but where histopathology of the PSMA-avid lesion was negative, where the PSA after targeted RT did not decrease by 50% or where post therapy imaging were not confirmatory. Regions where no PSMA-positive lesion was detected with additional confirmatory imaging or regions outside exclusively targeted RT with PSA decline were counted as true-negative (TN) regions. Patients with no histopathology, no further treatment or imaging, and therefore no composite standard of truth were not included in the final analysis (Figures 1 and 2).

### Statistical analysis

PET-positivity rate was defined as the proportion of rPC patients with PSMA-PET/CT findings clinically suspicious for or consistent with PC. Sensitivity (SE), specificity (SP), positive predictive value (PPV), negative predictive value (NPV), and positive and negative likelihood ratios (LR+/LR-) were calculated on a per region and per patient basis where CSOT was available to confirm TP, FP, TN, and FN lesions as described above. Receiver operator characteristic (ROC) and corresponding area under the curve (AUC) were computed. Statistical analyses were performed using Excel (Microsoft, Redmond, WA) and GraphPad Prism Version 6 (San Diego, CA). Confidence intervals were calculated according to Clopper-Pearson using the relationship between distribution and cumulative binomial distribution with limits of 100(1 − *α*) for *α* = 0.05 [32]. *P* values less than 0.05 were considered statistically significant.

## Results

### Rate of positive PET scans

**Fig. 3:**
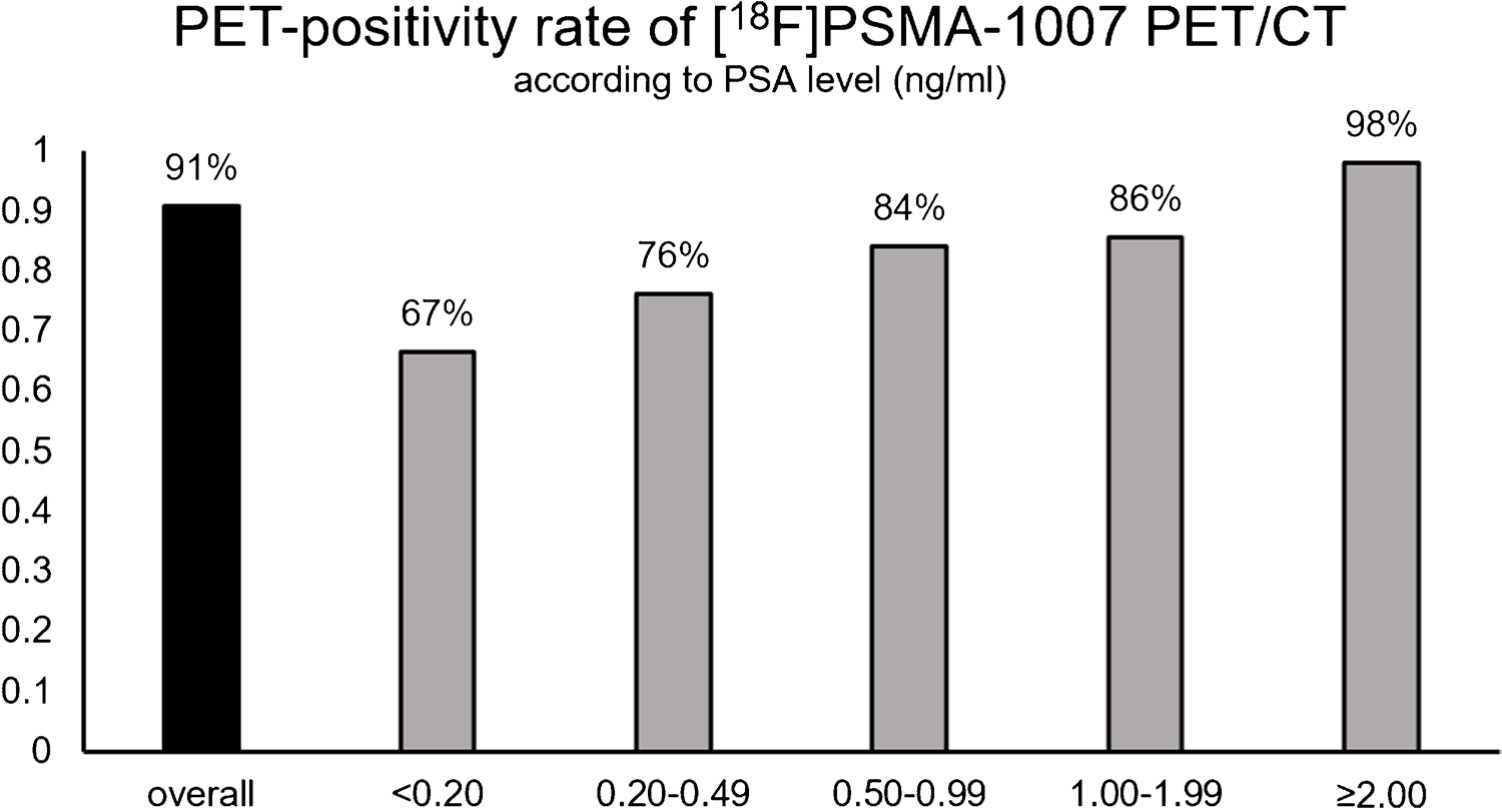
Frequency of all PSMA-positive PET/CTs in all patients with rPC (*n* = 177) and according to the PSA value before PET scan. Overall rate for a PSMA-positive scan in [^18^F]PSMA-1007 was 91%. PET-positivity rate increased with higher PSA levels (ng/ml) before PET-examination (< 0.2: 67%, 0.20–0.49: 76%, 0.50–0.99: 84%; 1.00–1.99: 86%; ≥ 2.00: 98%)

In all patients with rPC (*n* = 177) PSMA-PET/CT was positive in 161 patients (PET-positive rate = 91%). PET-positivity rate increased according to PSA level. At PSA < 2.0 ng/ml, 0.2–0.49 ng/ml, 0.50–0.99 ng/ml, 1.00–1.99 ng/ml, and ≥ 2.00 ng/ml, detection rates were 67%, 76%, 84%, 86%, and 98%, respectively (Figure 3).

### Patients of confirmatory follow-up

Of the included 177 patients with rPC, 152 patients had follow-up data available and 25 were lost to follow-up. Of the 152 patients with follow-up, 81 patients with rPC had follow-up data matching the CSOT criteria as outlined above. This could corroborate TP, FP, TN, and FN findings by confirmation or refutation on a per region basis with histopathological findings (*n* = 3), PSA decline (> 50%) (*n* = 23), or further imaging (*n* = 55). All patients’ treatments and characteristics are given in the Supplementary Material.

Of these 81 PSMA-PET/CTs in 73, at least one suspicious PSMA-avid lesion was detected, and therefore, the PET scan was rated as “positive”. This difference in PET-positivity rate for this subgroup to the overall positivity rate was not statistically significant (0.90 and 0.91 respectively, *p* = 0.21), implying no inherent bias in the subgroup of patients for whom confirmatory follow-up was available. In eight scans, no suspicious PSMA-avid lesion was reported. These scans were rated as “negative”. Follow-up data were confirmatory of PC in 65 scans of the 73 pathological-PET and in three of the eight non-pathological PET scans. Therefore, the patient-based sensitivity for [^18^F]PSMA-1007 in patients with rPC was 95.6% (95% confidence intervals (CI): 0.90–0.98).

### Region-based validation, sensitivity, specificity, PPV, and NPV

**Fig. 4:**
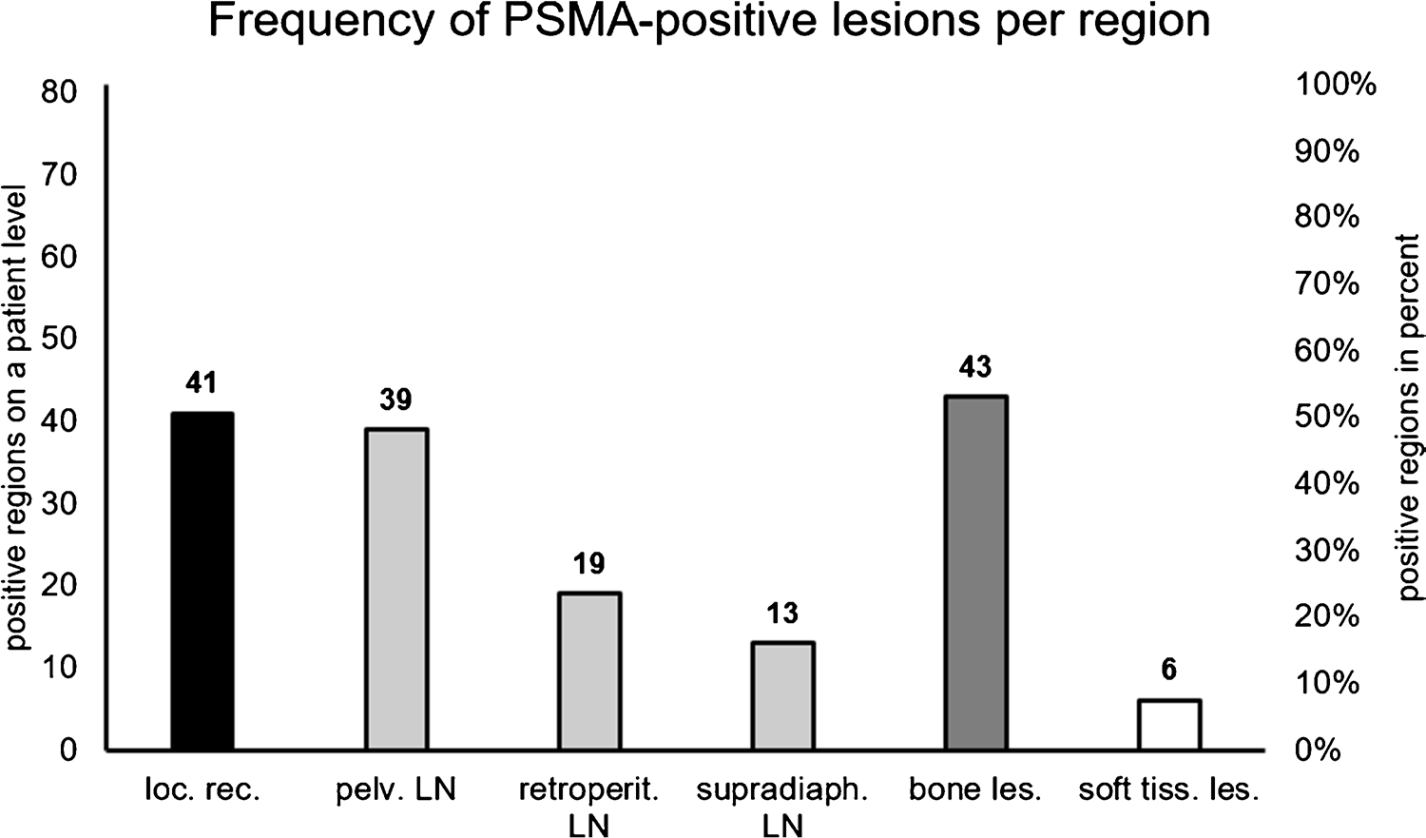
Frequency of PSMA-positive lesions per region in patients with CSOT follow-up (*n* = 81). Local recurrence (loc. rec.) was described in 41, pelvic (pelv.) lymph nodes (LN) in 39, retroperitoneal (retroperit.) LN in 19, supradiaphragmatic (supradiaph.) LN in 13, bone lesions (les.) in 43, and soft tissue lesions (soft tiss. les.) in 6 of 81 patients

CSOT confirmed or refuted PSMA-positive regions for recurrence of PC in 81/177 patients (45.7%). On a region basis, PSMA-positive lesions were detected in the prostatic fossa in 51%, in pelvic LN in 48%, in retroperitoneal LN in 23%, in supradiaphragmatic LN in 16%, in bones in 53%, and in other metastasis (soft tissue lesions) in 7% (Figure 4).

Overall sensitivity and specificity were 0.95 (CI: 0.90–0.98) and 0.89 (CI: 0.83–0.93), PPV and NPV were 0.86 (CI: 0.80–0.90) and 0.96 (CI: 0.92–0.98), and positive and negative likelihood ratio (LR+/LR-) were LR+: 8.71 (CI: 5.69–13.34) and LR−: 0.06 (CI: 0.03–0.12).

**Fig. 5:**
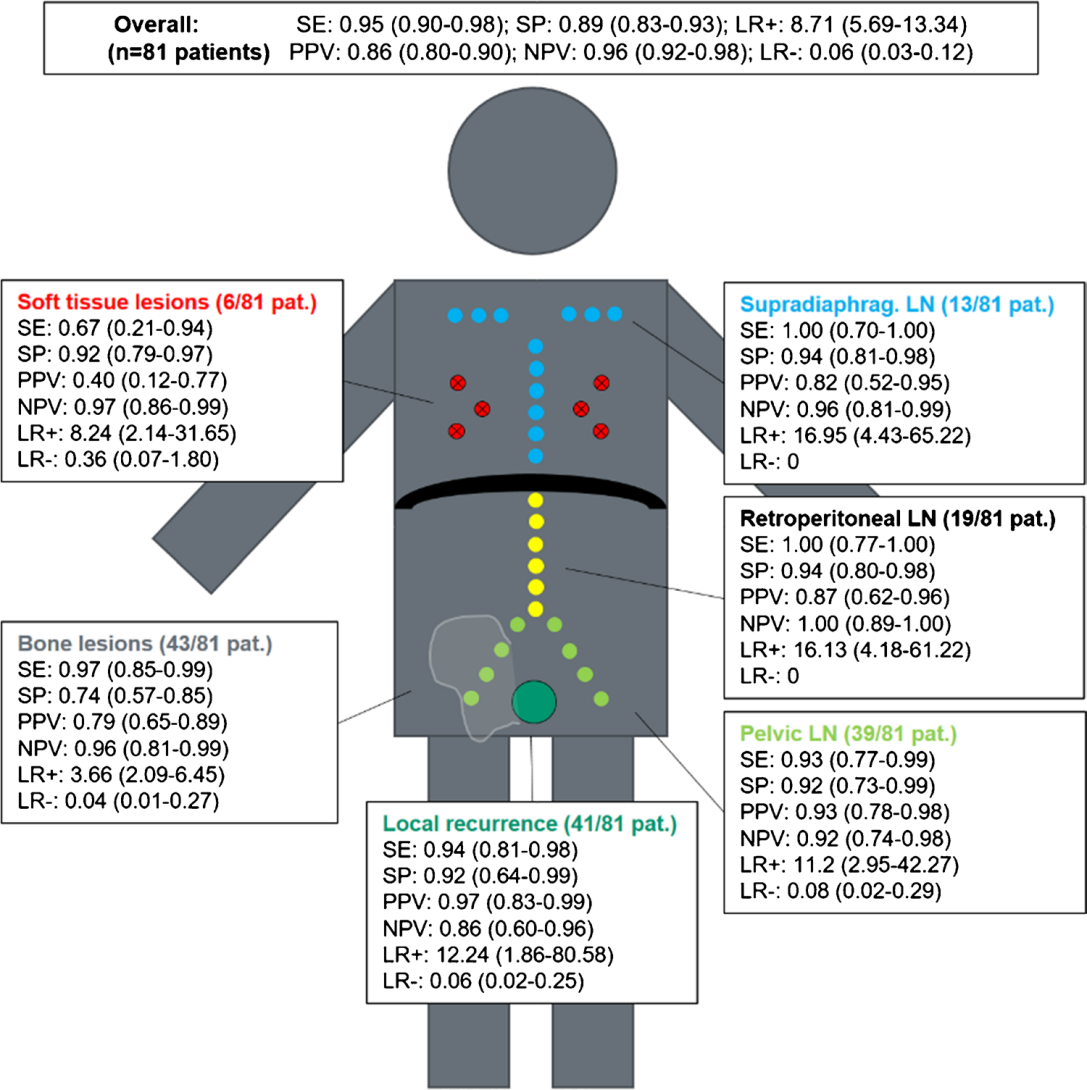
Sensitivity (SE), specificity (SP), positive predictive value (PPV), negative predictive value (NPV), and positive (LR+) and negative (LR−) likelihood ratio (LR) overall and on a region basis (local recurrence, pelvic lymph nodes (LN), retroperitoneal LN, supradiaphragmatic LN, bone lesions and soft tissue lesions) in patients (pat.) with rPC scanned with [^18^F]PSMA-1007 PET/CT

Sensitivity and specificity on a region basis were 0.94 (CI: 0.81–0.98) and 0.92 (CI: 0.64–0.99) for local recurrence, 0.93 (CI: 0.77–0.99) and 0.92 (CI: 0.0.73–0.99) for pelvic LN, 1.00 (CI: 0.77–1.00) and 0.94 (0.80–0.98) for retroperitoneal LN, 1.00 (CI: 0.70–1.00) and 0.94 (CI: 0.81–0.98) for supradiaphragmatic LN, 0.97 (CI: 0.85–0.99) and 0.74 (CI: 0.57–0.85) for bone lesions, and 0.67 (CI: 0.21–0.94) and 0.92 (CI: 0.79–0.97) for soft tissue lesions. PPV and NPV on a region basis were 0.97 (CI: 0.83–0.99) and 0.86 (CI: 0.60–0.96) for local recurrence, 0.93 (CI: 0.78–0.98) and 0.92 (CI: 0.74–0.98) for pelvic LN, 0.87 (CI: 0.62–0.96) and 1.00 (CI: 0.89–1.00) for retroperitoneal LN, 0.82 (CI: 0.52–0.95) and 0.96 (CI: 0.81–0.99) for supradiaphragmatic LN, and 0.79 (CI: 0.65–0.89) and 0.96 (CI: 0.81–0.99) for bone lesions. The small number of soft tissue lesions (*n* = 6) showed a PPV of 0.40 (0.12–0.77) and a NPV of 0.97 (CI: 0.86–0.99). Further details are given in Figure 5.

**Fig. 6:**
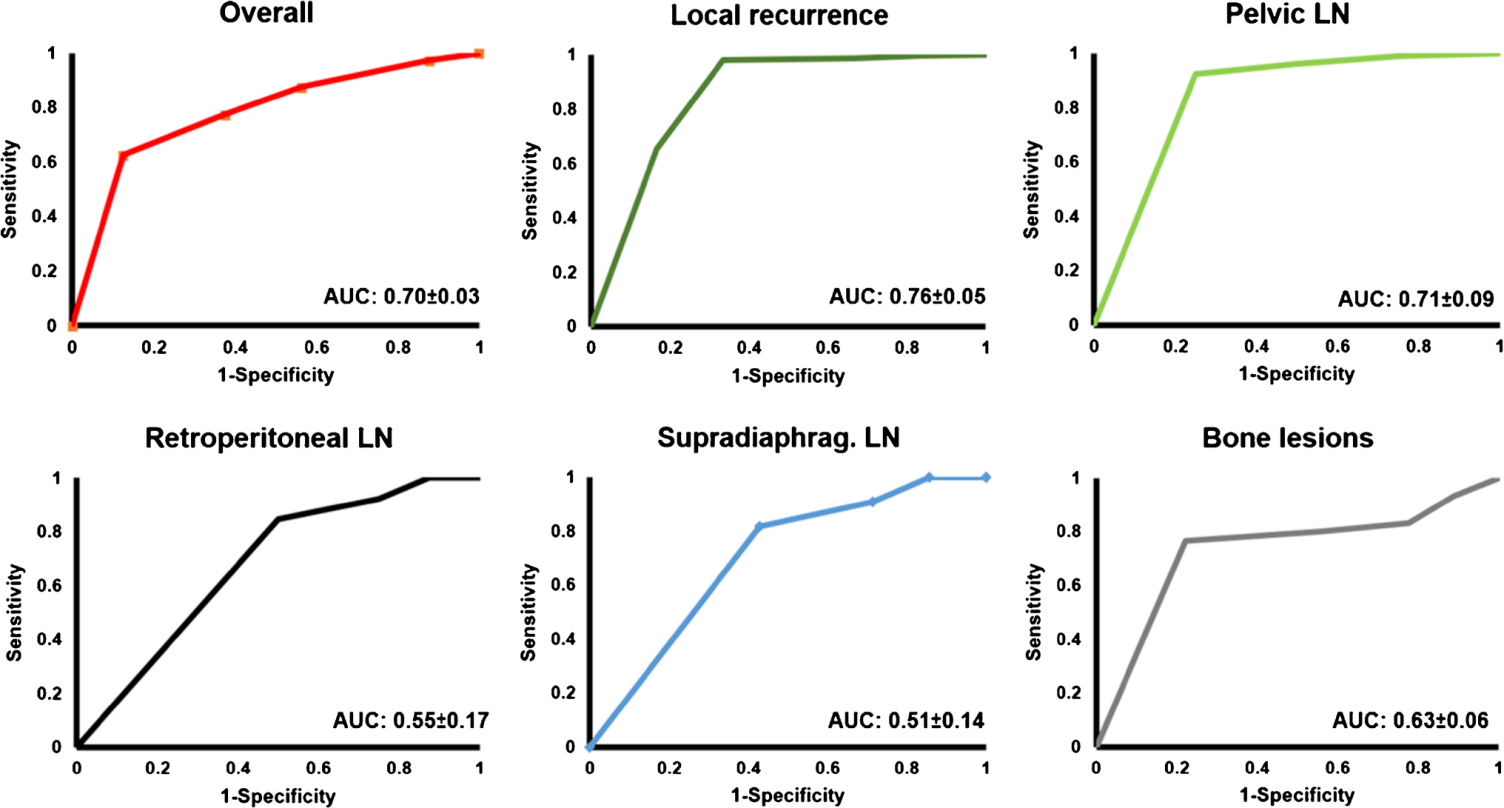
Receiver operating characteristic (ROC) and area under the curve (AUC) with 95% confidence interval for all lesions with CSOT follow-up on an overall and regional (local recurrence, pelvic lymph nodes (LN), retroperitoneal LN, supradiaphragmatic LN, and bone lesion) level. ROC and AUC for the soft tissue lesions which, with *n* = 6, were too small for analysis and not shown

ROC and corresponding area under the curves (AUC) were 0.70 ± 0.03 overall. For local recurrence, AUC was 0.76 ± 0.05, for pelvic LN 0.71 ± 0.09, for retroperitoneal LN 0.55 ± 0.17, and for supradiaphragmatic LN 0.51 ± 0.14. For bone lesions, the AUC was comparatively lower at 0.63 ± 0.06. Corresponding ROC curves are outlined in Figure 6.

## Discussion

[^18^F]PSMA-1007 was clinically first introduced in 2015 [33] and is now one of an increasing number of PSMA-radioligands. Despite the widespread implementation of [^18^F]PSMA-1007, there are currently very few evidence-based recommendations supporting its use [1]. One key attribute of a radiotracer is its clinical performance. In particular, where non-specific tracer uptake or false positives may lead to an incorrect staging, an analysis of the diagnostic accuracy is an essential factor when choosing which tracer to implement in a PSMA-PET programme. This study sought to assess the diagnostic performance for [^18^F]PSMA-1007 on a patient-level and a per region basis. In this retrospective analysis of 177 patients, we present the largest cohort of men undergoing [^18^F]PSMA-1007 for rPC with a confirmatory standard of follow-up [5, 14, 16, 26].

The overall PET-positivity rate of this tracer was high (91%) [1, 34, 35] and is in keeping with data reported by Rahbar et al. [5] (95%), Ahmadi Bidakhvidi et al. [36] (80%), and Giesel et al. [26] (81.3%). Similar to previous studies [26], we found increasing detection rates with increasing PSA (Figure 3), demonstrating high detection efficiency even in early rPC, and compares favourably with other tracers [1].

We find an overall sensitivity, specificity, and positive and negative predictive value in patients for whom CSOT follow-up was available (*n* = 81) of 95% (CI: 0.90–0.98), 89% (CI: 0.83–0.93), 86% (CI: 0.80–0.90), and 96% (CI: 0.92–0.98) respectively (Figure 5). These parameters are broadly in-keeping with those reported by Witkowska-Patena et al., who report SE, SP, PPV, and NPV of 100%, 94%, 66%, and 100% [15] for [^18^F]PSMA-1007 in a cohort of 40 patients with rPC. For nodal metastases, Sprute et al. report accuracy data following histological follow-up of [^18^F]PSMA-1007-avid lymph nodes in a mixed cohort of 96 patients with rPC and primary PC [14], with a SE and SP of 81.7% and 99.6% and a PPV and NPV of 92.4% and 98.9%, respectively [14]. These data are broadly similar to our findings for pelvic LN metastasis (SE: 93%, SP: 92%, PPV: 93%, and NPV: 92%) detected with [^18^F]PSMA-1007.

On per region basis, Watabe et al. report detection rates of [^18^F]PSMA-1007 for local recurrence (57.7%), pelvic LN (42.3%) and for bone lesions (15.4%) in a cohort of 28 patients with rPC, albeit without confirmatory follow-up [37]. We found similar results for local recurrence (50.6%) and pelvic LN (48.1%) detection rates but report a higher rate of PET-positive lesions in bones (53%), which is also in-keeping with other studies reporting a high rate of unspecific bone lesions in [^18^F]PSMA-1007 [38, 39]. In contrast to these previous studies, we are able to report, through a confirmatory standard of truth on a per region basis, data for the specific diagnostic performance for bone lesions. We demonstrate a more modest PPV for bone lesions (79%) compared to local recurrence (97%) or pelvic LN metastases (93%). Owing to this lower diagnostic performance in bone lesions, we posit that they are at risk of misclassification in rPC. In particular, FP findings could result in an incorrect staging or require further diagnostic or invasive tests, such as additional imaging or biopsy [11]. Further studies will be required to demonstrate the magnitude of the clinical impact of uncertain bone lesions.

One strength of our study was the high rate of follow-up available for our patients (86%). Through scrutiny of clinical imaging reports, multi-disciplinary team meeting minutes, and clinical records, we are able to demonstrate the performance of this radiopharmaceutical under clinical conditions in a large cohort of men undergoing PSMA-PET/CT in an academic nuclear medicine centre. Nevertheless, in common to all studies with rPC, follow-up data which can be used to verify imaging findings is not available for all patients. For example, a watchful waiting strategy might be chosen by the patient and his treating physicians. Using a CSOT including histopathology, PSA decline (> 50%) after targeted RT, and further imaging after the PSMA-PET showing response to system therapies, we were able to confirm or refute PSMA-PET findings. With a high number of consecutively screened patients with rPC (*n* = 177), 152 had follow-up data, of whom 81 had confirmatory CSOT; we report the largest cohort yet systematically investigated with confirmatory follow-up data for [^18^F]PSMA-1007. No significant difference in the PET-positivity rate between the subgroup with CSOT and those without was observed (*p* = 0.21), implying no bias in the patients with follow-up data available.

We highlight several weaknesses with our study. Our overall cohort size was limited by the introduction of the radioligand in our centre in Oct 2019 and the requirement to collect 12 months’ of follow-up. Although our cohort of patients with follow-up (*n* = 152) is not small, the relatively smaller number of supradiaphragmatic lymph nodes (*n* = 13) and solid organ metastases (*n* = 6) precluded an accurate analysis of diagnostic performance in these regions. Further studies, ideally of prospective design, are required to confirm these data. In order to reduce selection bias, patients were consecutively followed up. This heterogeneous cohort of patients at a variety of stages of rPC and prior treatment furnishes an overview of the radiotracer’s performance under routine clinical conditions. Further studies, for example, directed at early stages of recurrence should be performed, particularly since the PPV, which is dependent upon pre-test prevalence [11], might be yet lower in these cohorts.

## Conclusion

The known high detection rate for [^18^F]PSMA-1007 PET/CT in a cohort of men with rPC was confirmed with a high PET-positivity rate, with corresponding high sensitivity and NPV on a per region basis. Additionally, overall good diagnostic accuracy was observed with high PPV for local recurrences and pelvic lymph node metastasis. However, a lower PPV for bone lesions was observed, in-keeping with previous reports of high rates of non-specific bone uptake.

These results can assist in the interpretation of [^18^F]PSMA-1007, where knowledge of the diagnostic performance is a prerequisite to any proper evaluation of the post-test probability, i.e. the likelihood that a finding truly represents a lesion of rPC. The finding of a lower PPV for bone lesions in rPC suggests that these should be interpreted with caution, and if necessary prompt additional testing to reduce the risk of a FP.

## Supplementary Information

Below is the link to the electronic supplementary material.

Supplementary file1 (DOCX 74 KB)
