# Supplementary material for: Diagnostic accuracy of [18F]PSMA-1007 PET/CT in biochemical recurrence of prostate cancer
Source: Eur J Nucl Med Mol Imaging. 2022 Jan 24;49(7):2436–44. doi: 10.1007/s00259-022-05693-0 (PMC9165245; doi:10.1007/s00259-022-05693-0)
Supplement: Supplementary file 1 — Supplementary file1 (DOCX 74 KB) [file 259_2022_5693_MOESM1_ESM.docx]

# Supplementary material

## Initial treatment of all BCR-PC patients (n=177)

OP=radical prostatectomy and lymphadenectomy, RT= radiotherapy, ADT=androgen deprivation therapy

| OP | 135 | 76% |
| --- | --- | --- |
| RT | 8 | 5% |
| ADT | 18 | 10% |
| OP+RT | 2 | 1% |
| OP+ADT | 3 | 2% |
| RT+ADT | 5 | 3% |
| ADT+Chemotherapy+RT | 1 | 1% |
| Brachytherapy | 1 | 1% |
| OP+RT+ADT | 1 | 1% |
| Chemotherapy | 1 | 1% |
| no information | 2 | 1% |

## Detection rate


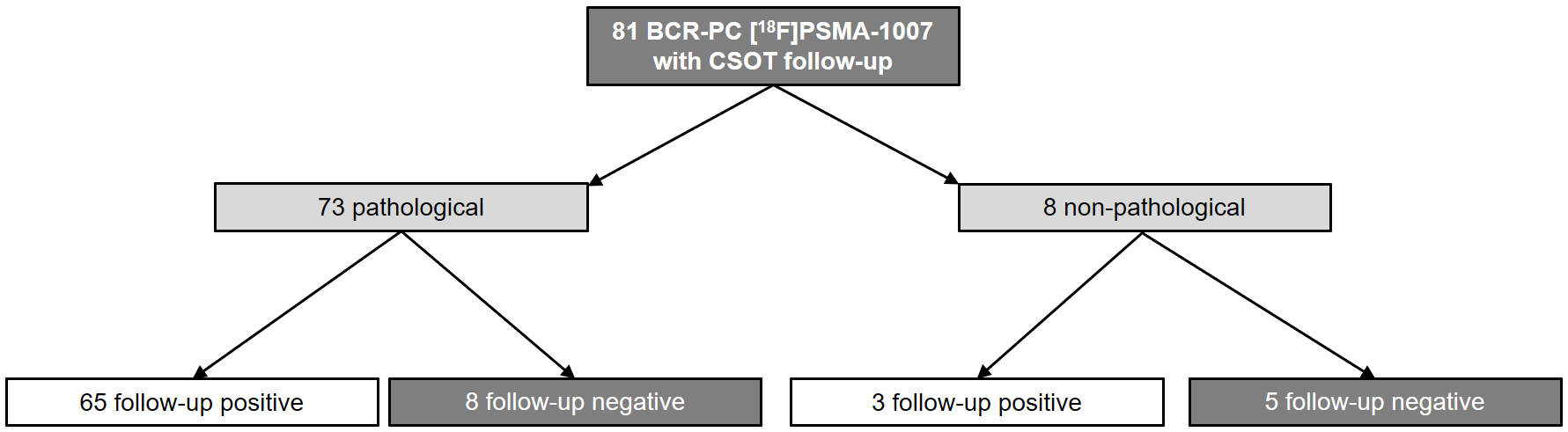


**Figure 1** Confirmation of pathological (PSMA-positive) and non-pathological (PSMA-negative) scans in patients with BCR-PC and CSOT follow up. Reported are 73/81 PSMA-positive (“pathological”) and 8/81 PSMA-negative (“non-pathological” scans). Follow up was positive for 68 patients. Detection rate was therefore 95.6% (65/68).

## Composite standard of truth follow up (n=81)

| Patient | Age | TNM | GS | PSA before PET | Histology | RT | PSA after treatment | Imaging |
| --- | --- | --- | --- | --- | --- | --- | --- | --- |
| 1 | 56 | T3b N0 M0 | 7 | 0.57 |  | yes (local and pelvic LN) |  | MRI local recurrent confirmed  Post therapy imaging (CTI) were not consistent with prior therapy imaging |
| 2 | 78 | T2c N0 | 7 | 10.7 |  | yes (local and pelvic LN) | 5.02 | CT progress of bone lesions |
| 3 | 78 | T2 N0 M0 | 6 | 10.8 |  |  |  | CT confirmation of bone lesions and pelvic LN;  Post therapy imaging (CT) were not consistent with prior therapy imaging |
| 4 | 80 |  | 8 | 25.5 |  |  | 0.095 | Post therapy imaging (CT) were not consistent with prior therapy imaging |
| 5 | 72 | T3 N0 M1 | 9 | 17.5 |  |  | 0.1 | CT confirmation of local recurrence and bone lesions |
| 6 | 67 | T3b N1 | 7 | 0.15 |  | yes (local and pelvic LN) | 0.01 | MRI confirmation of local recurrence;  Post therapy imaging (CTI) were not consistent with prior therapy imaging |
| 7 | 73 | T3b N1 M0 | 7 | 12.3 |  |  | 126.7 | CT confirmation of LN metastases and bone lesions |
| 8 | 75 | T2c N0 M0 | 7 | 0.9 |  |  | 0.1 | Post therapy imaging (CT) were not consistent with prior therapy imaging |
| 9 | 75 | T3a N0 M0 | 7 | 82.6 |  | yes (thoracal spine) | 18.5 | CT confirmation of bone lesions |
| 10 | 78 | T2c N0 M0 | 7 | 3.6 |  |  |  | CT confirmation of LN  Post therapy imaging (Ct) were not consistent with prior therapy imaging |
| 11 | 74 | T3a N0 M0 | 7 | 0.45 |  | yes (local and pelvic LN) | 0.44 |  |
| 12 | 77 | T4 N1 M1 |  | 27.2 |  |  |  | MRI and PSMA-PET confirmation local recurrence, pelvic and  retroperitoneal LN and bone lesions |
| 13 | 72 | T2a N0 M0 | 7 | 3300 |  |  |  | PSMA-PET confirmation of supradiaphragmatic LN and bone lesions |
| 14 | 74 | T3b N0 M0 | 7 | 45.1 |  |  | 72 | bone scan confirmation of bone lesions |
| 15 | 72 | T3b N0 | 9 | 0.19 |  |  |  | FDG-PET confirmation bone lesions |
| 16 | 87 | T2a N0 M0 | 9 | 9.6 |  | yes (local and pelvic LN) | 0.006 |  |
| 17 | 58 | T2 N1 | 7 | 0.17 |  | yes (local and pelvic LN) | 3.44 | MRI and PSMA-PET confirmation bone lesions |
| 18 | 64 |  | 7 | 9.81 |  |  |  | Post therapy imaging (PET) were not consistent with prior therapy imaging |
| 19 | 69 | T1 N0 M0 | 7 | 2.5 |  | yes (local and pelvic LN) | 0.42 | MRI confirmation of local recurrence |
| 20 | 52 | T2c N0 M0 |  | 2.1 |  |  |  | PSMA-PET negative |
| 21 | 51 | T3b N0 M0 | 6 | 0.34 |  |  | 0.39 | PSMA-PET progress of bone lesion |
| 22 | 83 | T2c N0 M0 | 7 | 1.51 |  |  | 1.89 | PSMA-PET confirmation local recurrence;  Post therapy imaging (CT) were not consistent with prior therapy imaging |
| 23 | 68 | T3b N1 M0 | 9 | 0.3 |  | yes (local and pelvic LN) | 0.1 |  |
| 24 | 76 | T3b N0 | 9 | 3.84 |  |  |  | bone scan confirmation of bone lesions |
| 25 | 65 |  |  | 1.28 | negative  (bone lesion) |  |  | Post therapy imaging (CT) were not consistent with prior therapy imaging |
| 26 | 65 | T3a N0 M0 | 7 | 1.79 |  |  | 2.46 | Post therapy imaging (MRI) were not consistent with prior therapy imaging |
| 27 | 62 | T2c N0 | 7 | 4.81 |  |  | 0.34 | PSMA-PET confirmation local recurrence, pelvic LN and bone lesions |
| 28 | 56 |  | 8 | 1.71 |  |  |  | MRI confirmation of pelvic LN |
| 29 | 61 | T3 N1 |  | 0.14 |  |  |  | PSMA-PET local recurrence and pelvic LN |
| 30 | 83 | T2b N1 M0 | 6 | 1.05 |  |  |  | MRI confirmation bone lesions |
| 31 | 67 | T3 | 8 | 2.6 |  |  |  | MRI confirmation local recurrence and pelvic LN |
| 32 | 73 |  |  | 462 |  |  |  | bone scan confirmation of bone lesions |
| 33 | 71 |  | 8 | 91.9 |  |  |  | PSMA-PET confirmation of all lesions |
| 34 | 63 |  | 7 | 2.6 |  |  |  | Post therapy imaging (MRI) were not consistent with prior therapy imaging |
| 35 | 66 | T2b N0 | 8 | 5.8 |  | yes (local and pelvic LN) | 0.1 | MRI confirmation bone lesions and pelvic LN |
| 36 | 79 | T3a N0 M0 | 8 | 0.6 |  | yes (local and pelvic LN) | 3.8 | PSMA-PET confirmation of retroperitoneal LN and bone lesions |
| 37 | 68 | T3b N0 M0 | 7 | 0.81 |  | yes (local and pelvic LN) |  | Post therapy imaging (MRI) were not consistent with prior therapy imaging |
| 38 | 63 | T4 N0 M0 | 9 | 1.3 |  |  | 6.42 | PSMA-PET confirmation of all lesions |
| 39 | 73 | T3a N0 M0 | 7 | 1.2 |  |  |  | MRI confirmation of pelvic LN and bone lesions |
| 40 | 75 | T3a N0 M0 | 8 | 1.4 |  |  |  | PSMA-PET negative |
| 41 | 59 | T2c | 7 | 0.76 |  |  | 1.45 | PSMA-PET negative |
| 42 | 74 | T3b N0 M0 | 8 | 0.61 |  | yes (local and pelvic LN) | 0.04 |  |
| 43 | 66 | T3b N1 M0 | 7 | 0.22 |  | yes (local and pelvic LN) | 0.003 |  |
| 44 | 69 | T3b N1 | 5 | 0.71 |  | yes (local and pelvic LN) | 0.64 | CT confirmation of soft tissue lesion |
| 45 | 61 | T1c N0 M1 | 9 | 2.1 |  |  |  | PSMA-PET confirmation of all lesions |
| 46 | 73 |  | 8 | 54.1 |  |  |  | PSMA-PET confirmation of all lesions |
| 47 | 74 | T3 N0 M0 | 7 | 2.1 |  | yes (local and pelvic LN) | 0.93 |  |
| 48 | 75 | T2b N0 M0 | 7 | 0.3 |  | yes (local and pelvic LN) | 0.1 |  |
| 49 | 80 | T3b N1 M1b | 7 | 48.9 | positive  (bone lesion) | yes (local and pelvic LN) |  | MRI confirmation of bone lesions |
| 50 | 74 | T3b N0 M0 | 9 | 1.9 |  | yes (local and pelvic LN) | 0.1 | Post therapy imaging (MRI) were not consistent with prior therapy imaging |
| 51 | 63 | T3b N1 M0 | 7 | 0.18 |  | yes (local and pelvic LN) | 0.15 |  |
| 52 | 68 | T2a N0 M0 | 7 | 0.37 |  | yes (local and pelvic LN) | 0.13 |  |
| 53 | 66 | T1c N0 M1b | 7 | 0.6 |  | yes (local and pelvic LN) |  | MRI and PSMA-PET confirmation of local recurrence |
| 54 | 77 |  | 7 | 2.7 |  |  |  | CT confirmation of bone lesion |
| 55 | 76 | T3b N0 M0 | 8 | 2.64 |  |  |  | Post therapy imaging (MRI) were not consistent with prior therapy imaging |
| 56 | 70 | T3a, N0, G2-3 | 7 | 4.76 |  |  | 15.49 | CT confirmation local recurrence and pelvic LN |
| 57 | 70 | T1c N0 M0 | 9 | 0.3 |  |  |  | MRI confirmation bone lesions |
| 58 | 68 | T3a, N0 |  | 0.47 | positive  (bone lesion) |  |  |  |
| 59 | 74 |  | 7 | 1.28 |  |  |  | MRI confirmation pelvic LN |
| 60 | 75 | T3b N1 M0 | 9 | 2.2 |  |  |  | CT confirmation local recurrence and pelvic LN |
| 61 | 73 | T3a N0 M0 | 7 | 0.3 |  | yes (local and pelvic LN) | 0.03 |  |
| 62 | 87 | T2 N0 M0 | 5 | 4.2 |  | yes (local and pelvic LN) |  | MRI confirmation local recurrence and bone lesions |
| 63 | 69 | T3b N0 | 7 | 6.4 |  | yes (local and pelvic LN) | 0.81 |  |
| 64 | 78 |  |  | 123 |  |  |  | PSMA-PET confirmation of local recurrence and bone lesions |
| 65 | 78 | T2 N0 M0 | 6 | 17.8 |  |  |  | MRI confirmation of local recurrence and bone lesions |
| 66 | 54 | T3b N1 M1 | 9 | 68.7 |  |  |  |  |
| 67 | 69 | T3a N1 M0 | 7 | 0.49 | positive  (bone lesion) | yes (bone lesion) | 0.56 |  |
| 68 | 73 | T2c N0 M0 | 7 | 0.5 |  |  |  | Post therapy imaging (MRI) were not consistent with prior therapy imaging |
| 69 | 66 | T3b N1 M0 | 9 | 518 |  |  |  | MRI and CT confirmation of bone lesions |
| 70 | 75 | T3b N1 Mx | 7 | 1.25 |  | yes (local and pelvic LN) | 0.003 |  |
| 71 | 77 | T2c N0 M0 | 7 | 1.35 |  | yes (local and pelvic LN) | 0.27 |  |
| 72 | 75 |  | 6 | 4.3 |  |  |  | MRI confirmation local recurrence and bone lesions |
| 73 | 71 | T3b N0 M0 | 10 | 0.38 |  | yes (local and pelvic LN) | 0.05 |  |
| 74 | 64 | T2c N0 | 7 | 3.39 |  | yes (local and pelvic LN) | 4.94 |  |
| 75 | 59 | T3b N1 M0 | 9 | 2.4 |  | yes (local and pelvic LN) | 1.41 |  |
| 76 | 79 | T3a N0 M0 | 7 | 1.93 |  | yes (local and pelvic LN) |  | MRI confirmation bone lesions |
| 77 | 77 | T1 N0 M0 | 7 | 11.3 |  | yes (local and pelvic LN) |  | MRI confirmation bone lesions |
| 78 | 60 | T3b N0 M0 | 7 | 9.66 |  | yes (local and pelvic LN) |  | PSMA-PET confirmation local recurrence and pelvic LN |
| 79 | 58 | T3 N1 M1 | 7 | 0.6 |  | yes (local and pelvic LN) |  | MRI confirmation bone lesions  Post therapy imaging (CT) were not consistent with prior therapy imaging |
| 80 | 63 | T3 N0 M1 | 9 | 11.3 |  |  |  | PSMA-PET confirmation local recurrence and pelvic, retro- and  supradiapharagmatic LN |
| 81 | 70 | T3b N0 M0 | 7 | 0.21 |  | yes (local and pelvic LN) | 0.24 |  |
